# Socioeconomic status across the early life course predicts gene expression signatures of disease and senescence

**DOI:** 10.1136/jech-2023-221812

**Published:** 2024-08-29

**Authors:** Cecilia Potente, Julien Bodelet, Hira Himeri, Steve Cole, Kathleen Harris, Michael Shanahan

**Affiliations:** 1 Erasmus School of Health Policy and Management, Erasmus Universiteit Rotterdam, Rotterdam, The Netherlands; 2 Lausanne University Hospital, Lausanne, Switzerland; 3 Jacobs Center for Productive Youth Development, University of Zurich, Zurich, Switzerland; 4 University of Zurich, Zurich, Switzerland; 5 University of California Los Angeles, Los Angeles, California, USA; 6 Department of Sociology, University of North Carolina at Chapel Hill, Chapel Hill, North Carolina, USA; 7 Carolina Population Center, University of North Carolina at Chapel Hill, Chapel Hill, North Carolina, USA

**Keywords:** Life course epidemiology, GENETICS, Health inequalities, BIOSTATISTICS, EPIDEMIOLOGY

## Abstract

**Background:**

Socioeconomic status (SES) is associated with many chronic diseases, indicators of senescence and mortality. However, the changing salience of SES in the prediction of adult health is not well understood. Using mRNA-seq abundance data from wave V of the National Longitudinal Study of Adolescent to Adult Health (Add Health), we examine the extent to which SES across the early life course is related to gene expression-based signatures for chronic diseases, senescence and inflammation in the late 30s.

**Methods:**

We use Bayesian methods to identify the most likely model of life course epidemiology (critical, sensitive and accumulation models) that characterises the changing importance of parental SES and SES during young (ages 27–30) and mid-adulthood (ages 36–39) in the prediction of the signatures.

**Results:**

For most signatures, SES is an important predictor in all periods, although parental SES or SES during young adulthood are often the most predictive. For three signatures (components of diabetes, inflammation and ageing), critical period models involving the exclusive salience of SES in young adulthood (for diabetes) or parental SES (for inflammation and ageing) are most probable. The observed associations are likely mediated by body mass index.

**Conclusion:**

Models of life course patterns of SES may inform efforts to identify age-specific mechanisms by which SES is associated with health at different points in life and they also suggest an enhanced approach to prediction models that recognise the changing salience of risk factors.

**WHAT IS ALREADY KNOWN ON THIS TOPIC**

- Parental and adult socioeconomic status (SES) are significantly associated with diseases later in life, yet their relative impact, in the context of life course models of epidemiology, is still to be determined.

**WHAT THIS STUDY ADDS**

- SES across the early life course is important for expression-based signatures of chronic diseases and senescence. Results point to the importance of sensitive period models: SES during adolescence and young and mid-adulthood all predict the signatures.

**HOW THIS STUDY MIGHT AFFECT RESEARCH, PRACTICE OR POLICY**

- Addressing health inequalities from the outset, beginning with the household of origin and extending into the early years of an individual’s own household, is crucial to mitigate long-term impacts and secure equitable health outcomes.

## Introduction

 Socioeconomic status (SES) during different life stages is strongly linked to adverse health outcomes, a phenomenon observed both within the USA and globally.^1^ However, the intricate social and biological mechanisms contributing to these negative health consequences remain to be fully elucidated. Parental SES (pSES) is likely associated with diseases in adulthood, including types 1 and 2 diabetes,^2^ some neoplasties,^3^ coronary heart disease,^4^ dementia and multimorbidities.^5^ Moreover, socioeconomic background is related to the treatment and management of many chronic diseases and also to risk factors for health, including common biomarkers.^6^

Nevertheless, the relative importance of SES at different times in the early life course is not well established. Existing evidence points to the long-lasting importance of childhood SES as it relates to differential exposure and vulnerability to psychosocial stressors^7^ as well as to physical stressors that reflect poor health-related habits.^8^ In turn, these psychosocial and physical stressors trigger gene expression patterns that, if chronically activated, lead to disease processes that unfold over many years of life.^9^ Indeed, evidence consistently reveals that low SES influences the molecular underpinnings of disease processes—as described with transcription and methylation data—that eventuate in poor health later in life largely via inflammatory and immune-related pathways.^10^,^12^

Life course epidemiology (LCE) proposes heuristic models to interpret studies of recurring risk and later health, including critical and sensitive period, accumulation, pathway and mobility models,^7^ distinctions that may be applied to the predictive salience of SES over decades of life. Findings from a recent scoping review are illustrative, suggesting that SES influences health according to a sensitive period or pathway model but not a critical period model. According to this scoping review, pSES predicts multimorbidity in later life, and this relationship may be partially accounted for by SES in adulthood (aSES). Accumulation models were not tested by any of the studies included in the review.^5^

Yet, the models that best correspond to findings about pSES in connection with aSES have often been limited.^5^ First, SES needs to be measured on multiple occasions,^13^ although extant studies typically focus on associations involving pSES alone^14^ or pSES while controlling aSES.^15^ Studies that only measure pSES are ambiguous in terms of the models of LCE since they cannot rule out any possibility. Studies that measure pSES and then aSES on only one occasion can rule out the critical period model and possibly pathways models but are otherwise ambiguous. Second, many studies rely on p values to determine if pSES and aSES are statistically significant predictors of health as tests of life course models, although the limitations of p values are well recognised.^16^

The present paper examines transcriptional patterns indicative of disease processes, ageing and inflammation using nationally representative, well-powered data from the National Longitudinal Study of Adolescent and Adult Health.^17^ The data include standard measures of parental and adult SES at three measurement occasions between roughly ages 12 and 42. The use of transcriptomic data is strategic because the sample is relatively young and healthy, in terms of diseases, but at risk for future health challenges. Thus, the study of the molecular underpinnings of disease provides potentially revealing data beyond what can be learnt from the study of disease states. Indeed, the prevalence of many common chronic conditions—including cardiovascular disease (CVD), rheumatoid arthritis, chronic obstructive pulmonary disease (COPD) and Alzheimer’s disease—increased markedly from the 40s onward, after the most recent wave of Add Health had been collected.^18^,^20^

An important mediating mechanism for the observed patterns of associations between SES and gene expression is body mass index (BMI), which is a proxy, although imperfect, for obesity. Recent meta-analyses have consistently shown that associations between SES and elevated inflammatory biomarkers are mediated by BMI,^21^ and BMI mediates associations between SES and several molecular mechanisms that underpin disease.^22^ BMI is not only due to genetic, psychological and metabolic causes but it is also deeply connected with social determinants of health.^23^ Life course theory,^7^ fundamental cause theory^24^ and health lifestyle theories^23^ have been used in previous research to describe the interrelation between social process and excessive weight gain. Indeed, Cockerham^23^ has highlighted how social determinants of obesity are an important facet to consider when studying health inequalities, especially early in the life course. Thus, we examine the extent to which BMI mediates the observed patterns.

We draw on Bayesian methods to assign probabilities that commonly studied models of LCE (ie, critical and sensitive periods, and accumulation) correspond to the data^25^ and, for sensitive period models, we identify the measurement occasions that are especially salient.^26^ Moreover, we test the mediating role of adult BMI in these associations. Identification of the most appropriate life course model for specific indicators of health is potentially valuable because inconsistent results in the study of SES and specific health outcomes may be reconciled by a life course approach.^27^ Moreover, the differing salience of SES across the life course may inform the creation of improved risk scores, identify possible strategic points for prevention and intervention efforts and guide the search for age-specific mediators.^28^

## Methods

Data come from waves I, IV and V of Add Health, a nationally representative sample of adolescents who have now entered mid-adulthood. SES is measured at three time points: wave I (pSES when individuals were largely between 14 and 17 years), wave IV (SES in young adulthood, at about ages 27–30) and wave V (SES in mid-adulthood largely at ages 36–39). SES at wave III was not considered because 37% of respondents were still in school and employment at this time of life may reflect relatively transient jobs.^29^ SES was measured as a standardised composite of the three indicators: education, income and socioeconomic index indicating occupational status. We draw on the mRNA-seq data of 3379 subjects (out of 4543 from whom mRNA data were collected) with complete information on the models’ variables. The principal source of missing data was pSES. The final mRNA sample is compared with the overall sample in table 1. Generally, the samples are roughly comparable, especially when considering the dispersions of variables.

**Table 1:** Descriptive information, Add Health

	mRNA subsample (N=3379)	Overall (N=12 300)
Sex		
Male	1364 (40.4%)	5334 (43.4%)
Female	2015 (59.6%)	6965 (56.6%)
Missing	0 (0%)	1 (0.0%)
Age at wave V		
Mean (SD)	37.3 (1.82)	37.6 (1.87)
Median (min, max)	37.0 (33.0, 42.0)	38.0 (33.0, 44.0)
Year of birth		
1974	1 (0.0%)	11 (0.1%)
1975	7 (0.2%)	69 (0.6%)
1976	164 (4.9%)	707 (5.7%)
1977	574 (17.0%)	2216 (18.0%)
1978	649 (19.2%)	2414 (19.6%)
1979	671 (19.9%)	2313 (18.8%)
1980	547 (16.2%)	1940 (15.8%)
1981	440 (13.0%)	1540 (12.5%)
1982	314 (9.3%)	1057 (8.6%)
1983	12 (0.4%)	33 (0.3%)
Race and ethnicity		
White Non-Hispanic	2270 (67.2%)	7205 (58.6%)
Black Non-Hispanic	535 (15.8%)	2389 (19.4%)
Asian Non-Hispanic	151 (4.5%)	714 (5.8%)
Other Non-Hispanic	37 (1.1%)	153 (1.2%)
Hispanic	386 (11.4%)	1839 (15.0%)
SES composite W1		
Mean (SD)	0.479 (2.34)	0.183 (2.34)
Median (min, max)	0.211 (−6.22, 4.89)	−0.00546 (−6.22, 4.89)
Missing	674 (19.9%)	3505 (28.5%)
Occupation scores W1		
Mean (SD)	42.7 (13.3)	41.6 (13.3)
Median (min, max)	36.8 (16.9, 60.9)	36.8 (16.9, 60.9)
Missing	0 (0%)	947 (7.7%)
Education W1		
High school	999 (29.6%)	4372 (35.5%)
Vocational	997 (29.5%)	3543 (28.8%)
College	707 (20.9%)	2293 (18.6%)
More than college	676 (20.0%)	1910 (15.5%)
Missing	0 (0%)	182 (1.5%)
Income W1		
Mean (SD)	52 600 (32700)	48 300 (32600)
Median (min, max)	45 000 (2500, 150 000)	45 000 (2500, 150 000)
Missing	674 (19.9%)	2933 (23.8%)
SES composite W4		
Mean (SD)	0.394 (2.21)	0.0693 (2.27)
Median (min, max)	0.429 (−7.23, 4.79)	0.0398 (−7.27, 4.79)
Missing	0 (0%)	2227 (18.1%)
Occupation scores W4		
Mean (SD)	51.9 (21.6)	49.2 (21.5)
Median (min, max)	53.8 (10.6, 92.8)	46.5 (10.6, 92.8)
Missing	0 (0%)	1644 (13.4%)
Education W4		
High school	483 (14.3%)	2253 (18.3%)
Vocational	1408 (41.7%)	4747 (38.6%)
College	870 (25.7%)	2372 (19.3%)
More than college	618 (18.3%)	1538 (12.5%)
Missing	0 (0%)	1390 (11.3%)
Income W4		
Mean (SD)	66 000 (38100)	63 700 (38200)
Median (Min, Max)	62 500 (2500, 150 000)	62 500 (2500, 150 000)
Missing	0 (0%)	2011 (16.3%)
SES composite W5		
Mean (SD)	0.492 (2.31)	0.0494 (2.39)
Median (min, max)	0.635 (−6.39, 4.59)	0.0177 (−6.39, 4.59)
Missing	0 (0%)	618 (5.0%)
Occupation scores W5		
Mean (SD)	54.8 (22.1)	53.4 (22.2)
Median (min, max)	58.6 (10.6, 93.7)	55.1 (10.6, 93.7)
Missing	0 (0%)	7933 (64.5%)
Education W5		
High school	426 (12.6%)	2310 (18.8%)
Vocational	1265 (37.4%)	4969 (40.4%)
College	833 (24.7%)	2566 (20.9%)
More than college	850 (25.2%)	2433 (19.8%)
Missing	5 (0.1%)	22 (0.2%)
Income W5		
Mean (SD)	91 000 (55 900)	84 200 (56 800)
Median (min, max)	87 500 (2500, 200 000)	62 500 (2500, 200 000)
Missing	0 (0%)	175 (1.4%)

SESsocioeconomic status

Education is the maximum grade completed by either parent, divided into four categories: high school and less, vocational, college, and more than college. Parental income is the gross household income, log-transformed and recoded in 12 categories for comparability with adult income. Parental occupation represents the highest socioeconomic index score of parents’ jobs. In waves IV and V, education is reported as the highest self-reported years of education in the same four categories. Income is the gross family income, reported on an ordinal scale with values representing midpoints of 12 categories. Occupation represents the socioeconomic index score of the current job^30^ (see online supplemental appendix 1, table S1 and online supplemental dataset S1 for details).

Transcriptomic profiles of consenting participants were collected during wave V of the Add Health Study (2016–2017) via an intravenous blood draw. Detailed information on the study design, interview procedures, consent procedures, demographic assessments, collection, sequencing and quality control of the blood sample, and derivation of the analytical samples is reported in previous studies.^11^ Genes with low counts were excluded from the analysis. Normalisation of the raw mRNA-seq counts is based on weighted trimmed mean of log expression ratios (Trimmed Mean of the M-values (TMM) normalisation) and we also corrected for batch effects using an empirical Bayes framework (see online supplemental appendix 2 for details on implementation using R).

We selected 13 disease and senescence signatures reflecting common chronic conditions in the American population and, for each signature, used sparse principal component analysis to reduce dimensionality; the optimal number of sparse principal components (PCs) was identified. The signatures were derived from out-of-sample genome-wide and expression-wide association studies (see online supplemental table S2). The optimal number of principal components was determined by inspecting scree plots, and the weights from the PCs were reported in an Excel file (online supplemental file). Thus, we obtain several summary measures for each gene set signature, reflecting the number of PCs (in parenthesis): CVD (4), lupus (5), colorectal cancer (4), rheumatoid arthritis (5), asthma (6), hypertension (3), aortic aneurysm (3), COPD (3), diabetes (6), inflammation (4), Alzheimer (7) and senescence (4). The sparse PCs loadings for each gene set signature are available in online supplemental dataset S2.

The assignment of the most appropriate model of LCE—critical, accumulation and sensitive models—is based on a two-step Bayesian procedure. We first use the relevant life course model^31^ and then choose the most descriptive life course models using the sequential partitioning test (SPT) procedure.^26^ In the first step, a regression model estimates the association between SES across all measurement occasions and each PC (ie, a global association), as well as the relative weights (summing to 1) associated with each measurement occasion. We simultaneously model all PCs within a Bayesian hierarchical framework. This approach addresses the multiple comparison issues.^32^ Moreover, one of the advantages of the Bayesian framework is the possibility of directly modelling missing data.^33^ We use the Bayesian imputation procedure to impute missing parental income, a major source of missingness (see table 1). For the imputation, we used 21 variables to predict parental income (see methodological details in online supplemental appendix 3).

In the second step, the probability of each life course model is evaluated using the range statistics (the maximum weight minus the minimum weight) which lies on the interval (0, 1).^26^ A range statistic close to 0 or 1 indicates an accumulation and critical model, respectively, and a sensitive model is otherwise indicated. We use the regions of the practical equivalence approach and partition the unit interval as (0, 0.15), (0.15, 0.85), (0.85, 1) to indicate accumulation, sensitive period model and critical period, respectively. For example, estimated weights equalling 0.40, 0.25 and 0.35 would fall into the accumulation interval (ie, 0.40−0.25=0.15). The probability of each model is then calculated as the proportion of posterior estimates falling into intervals that correspond to the three models. For sensitive period results, the simplex representing the three weights is partitioned until the most credible solution is reached (for full details see, Chumbley *et al*^26^). Controls include birth year, biological sex, race/ethnicity, region and sample-specific quality control measures for mRNA.

Finally, we consider the extent to which BMI mediates observed patterns given the prominent role of BMI in past research.^11 25^ The direct and average causal mediated effects (ACMEs) are estimated in a counterfactual framework. For these models, SES scores reflect the sum of SES at the three time periods weighted by their respective weights and the total is further weighted by the global association.

## Results

Table 2 presents the posterior probabilities for accumulation, sensitive and critical period life course models for which we observe a credible global association with total early life and adulthood SES (ie, when the 95% credible interval of the global association did not contain 0). Of 54 disease signature PCs, 17 showed a credible association with total early life and adulthood SES. For example, the cardiovascular PC1 gene set is credibly associated with total early life and adulthood SES, with a sensitive period model being most probable (p=0.80), followed by critical period (p=0.18), and the accumulation model is quite unlikely (p=0.02). Table 2 shows that accumulation models always are very unlikely, and in most instances, sensitive period models are clearly most likely.

**Table 2:** Probability of regions of practical equivalence for three broad life course models based on Chumbley *et al*^26^

Signatures	Accumulation	Sensitive	Critical
CVD (PC1)	0.02	0.80	0.18
Lupus (PC5)	0.01	0.66	0.33
Colorectal (PC3)	0.03	0.90	0.08
RA (PC1)	0.00	0.62	0.38
RA (PC4)	0.01	0.61	0.38
RA (PC5)	0.03	0.83	0.14
Asthma (PC2)	0.01	0.72	0.26
Asthma (PC3)	0.01	0.71	0.28
Diabetes (PC3)	0.01	0.66	0.34
Diabetes (PC5)	0.00	0.45	0.54
Inflammation (PC2)	0.00	0.43	0.57
Inflammation (PC3)	0.02	0.75	0.23
Inflammation (PC4)	0.01	0.75	0.24
Alzheimer (PC2)	0.01	0.69	0.30
Alzheimer (PC6)	0.01	0.86	0.13
Ageing (PC2)	0.00	0.49	0.51
Ageing (PC4)	0.00	0.71	0.28

CVDcardiovascular diseasePCprincipal componentRArheumatoid arthritis

In the three instances where the critical period model is most likely—diabetes (PC5), inflammation (PC2) and ageing (PC2)—the results are ambiguous because the probabilities associated with the sensitive period models are comparably high. Nevertheless, the exclusive salience of SES in young adulthood for diabetes (PC5) and pSES for inflammation (PC2) and ageing (PC2) is the most probable among the options.

Table 3 reports the posterior probability of the most credible ranking for SES in different life course periods for the sensitive period models identified in table 2 for which a decisive posterior probability from the partitioning procedure was observed. Table 3 suggests three conclusions. First, in all instances, SES at one period of life is more salient than at the other two measurement occasions. This pattern may appear to correspond to a critical period pattern but, given the results reported in table 2, it indicates that one period is more salient than the other two periods, and the magnitude of the latter two cannot be distinguished but they are important predictors. Second, SES in young adulthood is the most salient period for about half of the PCs. Third, pSES was most salient for the other PCs, with young adulthood being important but indistinguishable from mid-adulthood.

**Table 3:** Ranking measurement occasions by their importance (ie, their relative magnitude) for PCs (with credible lifetime SES coefficients)

Signatures	Ranking	Posterior probability
Lupus (PC5)	SES in young adulthood>parental SES and SES in mid-adulthood	0.85
CVD (PC1)	Parental SES>SES in young and mid-adulthood	0.62
Colorectal (PC3)	Parental SES>SES in young and mid-adulthood	0.64
RA (PC1)	Parental SES>SES in young and mid-adulthood	0.94
RA (PC4)	SES in young adulthood>parental SES and SES in mid-adulthood	0.82
RA (PC5)	SES in young adulthood>parental SES and SES in mid-adulthood	0.44
Asthma (PC2)	SES in young adulthood>parental SES and SES in mid-adulthood	0.66
Asthma (PC3)	Parental SES>SES in young and mid-adulthood	0.76
Diabetes (PC3)	Parental SES>SES in young and mid-adulthood	0.81
Diabetes (PC5)	SES in young adulthood>parental SES and SES in mid-adulthood	0.87
Inflammation (PC2)	Parental SES>SES in young and mid-adulthood	0.92
Inflammation (PC3)	SES in young adulthood>parental SES and SES in mid-adulthood	0.63
Inflammation (PC4)	SES in young adulthood>parental SES and SES in mid-adulthood	0.83
Alzheimer (PC2)	SES in mid-adulthood>parental SES	0.62
Alzheimer (PC6)	Parental SES and SES in young adulthood>SES in mid-adulthood	0.86
Ageing (PC2)	Parental SES>SES in mid-adulthood	0.96
Ageing (PC4)	SES in young adulthood>parental SES and SES in mid-adulthood	0.86

CVDcardiovascular diseasePCsprincipal componentsRArheumatoid arthritisSESsocioeconomic status

Finally, we examined the extent to which BMI might mediate associations between weighted lifetime SES and the PCs. Results in table 4 indicate credible mediation of BMI (ie, when the 95% credible interval of the ACME does not contain 0 for most of the 17 PCs (as reported in table 1). The proportion mediated varies depending on the gene set, ranging from 52% for lupus (PC5) to 6% for asthma (PC3), but for many signatures about 20%–30% of the association may be mediated by BMI. These results suggest the possibility that lifestyle factors such as BMI may partially mediate the observed association between early-life and adulthood SES and gene expression.

**Table 4:** Decomposition of the weighted total effects in average direct effect (ADE) and average causal mediated effects (ACME) with credible interval for each of the PCs

Signatures	ADE	ACME	(Credible interval)	Proportion mediated
CVD (PC1)	0.016	0.008	(0.005, 0.012)	0.331
Lupus (PC5)	0.015	0.016	(0.012, 0.021)	0.518
Colorectal (PC3)	0.033	0.009	(0.005, 0.013)	0.208
RA (PC1)	0.022	0.009	(0.005, 0.012)	0.282
RA (PC4)	0.024	0.005	(0.003, 0.008)	0.166
RA (PC5)	0.022	0.006	(0.003, 0.010)	0.219
Asthma (PC2)	0.034	0.006	(0.003, 0.009)	0.143
Asthma (PC3)	0.025	0.002	(−0.001, 0.005)	0.061
Diabetes (PC3)	0.011	0.006	(0.003, 0.009)	0.343
Diabetes (PC5)	0.015	0.009	(0.006, 0.013)	0.383
Inflammation (PC2)	−0.021	−0.005	(−0.008, –0.002)	0.185
Inflammation (PC3)	0.015	0.006	(0.004, 0.009)	0.296
Inflammation (PC4)	0.030	0.014	(0.010, 0.018)	0.311
Alzheimer (PC2)	−0.028	0.000	(−0.003, 0.002)	0.012
Alzheimer (PC6)	0.022	0.009	(0.006, 0.013)	0.291
Ageing (PC2)	0.027	0.003	(0.001, 0.006)	0.110
Ageing (PC4)	0.039	0.012	(0.008, 0.016)	0.231

CVDcardiovascular diseasePCprincipal componentRArheumatoid arthritis

## Discussion

This paper examines the changing salience of SES across the early life course—spanning adolescence to mid-adulthood—in the prediction of mRNA-seq signatures for common, chronic diseases, senescence and inflammation among participants in a large, diverse sample. Previous research suggests the importance of pSES in the prediction of adult disease, but studies increasingly focus on whether SES’s predictive power changes over decades of life.^27 34^ Such information may be helpful to improve risk models that predict health outcomes by providing weights for SES on multiple occasions in life as opposed to current practice, which pays little attention to the changing salience of repeated exposures to risk (eg, the Framingham risk score to predict cardiovascular events^35^ and the Cardiovascular Risk Factors, Ageing and Incidence of Dementia score to predict future dementia.^36^ Also, such research provides clues about change and stability in mediating mechanisms that link status with disease, and they also suggest strategic ages for intervention and prevention.

Indeed, the results point to the importance of sensitive period models in predicting PCs related to CVD, colorectal cancer, rheumatoid arthritis, asthma, inflammation, Alzheimer and ageing. (Several signatures were best predicted by a critical period model, but they were all characterised by considerable uncertainty.) The predominance of sensitive period models is noteworthy for four reasons. First, although it is true that sensitive period models are a ‘catch-all’ category that refers to any pattern that is not accumulation or critical period, these latter two possibilities are tested using regions of practical equivalence, meaning that a considerable range of weights would qualify as either model.

Second, the omnibus test identifies sensitive period models, and the SPT identifies SES in one period of life as especially salient and the other two occasions as important but indistinguishable in their associated weights. This pattern may also be regarded as a ‘relaxed accumulation model’, according to which SES at all measurement occasions is consequential, but to varying degrees. Thus, SES during adolescence and young and mid-adulthood all predict the signatures, and one occasion is most predictive.

Third, none of the sensitive period models posits that the measurement occasion that is contemporaneous with the signature is most predictive. Contemporaneous SES could reasonably be expected to predict health because it affects one’s immediate living conditions, including such factors as access to healthcare, wholesome foods, exercise facilities, a clean environment, and, indeed, available strategies to cope with a changing climate. Such factors should be especially relevant to mRNA-seq abundance levels, which are somewhat transient, at least at the level of specific genes. Nevertheless, pSES or SES during young adulthood is decidedly most salient in the prediction of the signatures.

Finally, the results raise the possibility of different mechanisms or the changing importance of the same mechanisms across the life course. Such a view has been adopted, for example, by the Lancet Commission on Dementia, which documented that, based on the best available evidence, mechanisms that increase the odds of dementia change considerably from young to mid-adulthood.^37^ Future research would ideally investigate whether inequalities in senescence and chronic disease reflect mechanisms that change across the life course and, indeed, our modelling strategy can accommodate multiple risk factors. Given that people’s role configurations—involving various types of students, intimate relations and work—change considerably, their stress exposures and the resources needed to address them likely change as well.

Most of the observed associations are possibly mediated by BMI. Higher BMI is associated with chronic inflammation caused by adipose tissue which might influence dysregulation in gene expression. Moreover, BMI is responsible for many deaths worldwide through its involvement in the genesis of many diseases, such as CVD, type 2 diabetes and various cancers. Therefore, it is important to better understand the mediating role of BMI in the association between life course SES and gene expression.

Several limitations should be noted. First, the data and methods do not allow for causal inference but rather the results should be construed as multivariate descriptions. Although there are strategies to identify the effects of education or income on health (eg, family-based fixed effect models or Mendelian randomisation), the identification of unique SES effects at multiple time points spanning many decades of life remains a vexing problem. Second, the signatures are based on results from genome-wide and expression-wide association studies that are from predominantly Caucasian samples (from the USA, the UK and Iceland). Yet results based on such samples may obscure ancestry-distinct associations between genetic variation and phenotypes,^38^ meaning that the signatures may not be equally applicable to all ancestral groupings that are represented in Add Health. Third, tests of life course models of epidemiology—for example, whether the data best correspond to a sensitive period model—assume that substantive conclusions are not sensitive to changes in the timing or number of measurement occasions. Third, our associational study seeks to determine correlations between SES at different time points and health, but we are not able to answer the question of whether it is SES that influences health or vice-versa. Finally, although the results point to sensitive period models for most signatures, the mediational model only examines BMI in wave V. Although such a specification is of interest, evidence suggests that BMI at different points in life may have independent, salient associations with at least some indicators of adult health,^39 40^ including expression-based signatures.^25^

Nevertheless, the present study provides evidence that SES across the early life course is important to the prediction of the expression-based signatures for chronic diseases and senescence, and thus suggests the importance of reducing inequalities in health by targeting mechanisms associated with SES in the household of origin but also in the first years of one’s own household. Based on the present results and previous research, BMI is likely to be a prominent source of explanation, although SES-based mechanisms that are age-specific remain to be explored. A significant challenge when investigating health inequalities in adult chronic diseases is that outcomes typically occur in later life, but the underlying processes are operative for decades.^13^ Gene expression data provided us with a novel window into predisease patterns because they reveal evidence of chronic disease mechanisms already in young adulthood. Our results highlight the sensitive period as the most likely LCE model confirmed by the data. These results are in line with previous literature as demonstrated by a recently published meta-analysis on life course socioeconomic conditions and multimorbidity in old age.^5^ Further research should investigate possible interventions in early life aimed at decreasing the burden of diseases later in life.

## supplementary material

10.1136/jech-2023-221812 online supplemental file 1

10.1136/jech-2023-221812 online supplemental file 2

10.1136/jech-2023-221812 online supplemental file 3

## Data Availability

Data are available in a public, open access repository. Data are available on reasonable request. Data may be obtained from a third party and are not publicly available.
