# Supplementary material for: Socioeconomic status across the early life course predicts gene expression signatures of disease and senescence
Source: J Epidemiol Community Health. 2024 Aug 29;78(12):e221812. doi: 10.1136/jech-2023-221812 (PMC11671950; doi:10.1136/jech-2023-221812)
Supplement: online supplemental file 1 [file jech-78-12-s001.pdf]

SOC2000	SOC2010	Census Titles	Occupation Scores
11-1011	10	Chief executives	77,4
11-1021	20	General and operations managers	68,6
11-2011	40	Advertising and promotions managers	73,1
11-2021	50	Marketing and sales managers	73,9
11-2022	50	Marketing and sales managers	73,9
11-2031	60	Public relations and fundraising managers	82,4
11-3011	100	Administrative services managers	64,9
11-3021	110	Computer and information systems managers	83,8
11-3031	120	Financial managers	69,3
11-3041	135	Compensation and benefits managers	73,2
11-3049	136	Human resources managers	68,6
11-3042	137	Training and development managers	73,9
11-3051	140	Industrial production managers	68,0
11-3061	150	Purchasing managers	71,6
11-3071	160	Transportation, storage, and distribution managers	52,5
11-9011	205	Farmers, ranchers, and other agricultural managers	39,9
11-9021	220	Construction managers	59,1
11-9031	230	Education administrators	75,5
11-9032	230	Education administrators	75,5
11-9033	230	Education administrators	75,5
11-9039	230	Education administrators	75,5
11-9041	300	Architectural and engineering managers	89,1
11-9051	310	Food service managers	41,0
11-9061	325	Funeral service managers	67,7
11-9071	330	Gaming managers	52,1
11-9081	340	Lodging managers	50,3
11-9111	350	Medical and health services managers	71,8
11-9121	360	Natural sciences managers	86,9
11-9131	400	Postmasters and mail superintendents	67,7
11-9141	410	Property, real estate, and community association managers	54,6
11-9151	420	Social and community service managers	67,8
11-9199	430	Managers, all other	67,7
13-1011	500	Agents and business managers of artists, performers, and athletes	60,2
13-1021	510	Buyers and purchasing agents, farm products	48,8
13-1022	520	Wholesale and retail buyers, except farm products	50,0
13-1023	530	Purchasing agents, except wholesale, retail, and farm products	59,9
13-1031	540	Claims adjusters, appraisers, examiners, and investigators	59,8
13-1032	540	Claims adjusters, appraisers, examiners, and investigators	59,8
13-1041	565	Compliance officers	69,4
13-1051	600	Cost estimators	62,4
13-1079	630	Human resources workers	65,3
13-1071	640	Employment	62,4
13-1072	640	Compensation, benefits, and job analysis specialists	62,4
13-1073	650	Training and development specialists	64,2
13-1081	700	Logisticians	61,9
13-1061	710	Management analysts	78,3
13-1111	710	Emergency Management analysts	78,3

13-1121	725	Meeting, convention, and event planners	62,1
19-3021	735	Market research analysts and marketing specialists	75,6
13-1199	740	Business operations specialists, all other	63,7
13-2011	800	Accountants and auditors	76,3
13-2021	810	Appraisers and assessors of real estate	62,6
13-2031	820	Budget analysts	77,3
13-2041	830	Credit analysts	65,6
13-2051	840	Financial analysts	81,9
13-2052	850	Personal financial advisors	79,3
13-2053	860	Insurance underwriters	66,2
13-2061	900	Financial examiners	76,5
13-2071	910	Credit counselors and loan officers	62,1
13-2072	910	Credit counselors and loan officers	62,1
13-2081	930	Tax examiners and collectors, and revenue agents	60,3
13-2082	940	Tax preparers	57,0
13-2099	950	Financial specialists, all other	64,4
15-1011	1005	Computer and information research scientists	84,3
15-1051	1006	Computer systems analysts	80,7
15-1011	1007	Information security analysts	77,2
15-1021	1010	Computer programmers	81,0
15-1031	1020	Software developers, applications and systems software	87,9
15-1032	1020	Software developers, applications and systems software	87,9
15-1051	1020	Software developers, applications and systems software	87,9
15-1041	1050	Computer support specialists	64,5
15-1061	1060	Database administrators	77,0
15-1071	1105	Network and computer systems administrators	74,8
15-1081	1106	Computer network architects	83,4
15-1099	1107	Computer occupations, all other	70,5
15-2011	1200	Actuaries	93,7
15-2021	1210	Mathematicians	85,0
15-2031	1220	Operations research analysts	80,2
15-2041	1230	Statisticians	85,0
15-2099	1240	Miscellaneous mathematical science occupations	85,0
17-1011	1300	Architects, except naval	84,0
17-1012	1300	Architects, except naval	84,0
17-1022	1310	Surveyors, cartographers, and photogrammetrists	77,9
17-2011	1320	Aerospace engineers	89,3
17-2031	1340	Biomedical engineers	86,1
17-2041	1350	Chemical engineers	87,6
17-2051	1360	Civil engineers	85,2
17-2061	1400	Computer hardware engineers	81,5
17-2071	1410	Electrical and electronics engineers	86,5
17-2072	1410	Electrical and electronics engineers	86,5
17-2081	1420	Environmental engineers	85,5
17-2111	1430	Industrial engineers, including health and safety	81,6
17-2112	1430	Industrial engineers, including health and safety	81,6
17-2121	1440	Marine engineers and naval architects	82,9
17-2131	1450	Materials engineers	83,1

17-2141	1460	Mechanical engineers	83,8
17-2151	1500	Mining and geological engineers, including mining safety engineers	84,3
17-2161	1510	Nuclear engineers	86,1
17-2171	1520	Petroleum engineers	84,3
17-2199	1530	Engineers, all other	86,1
17-3011	1540	Drafters	62,7
17-3012	1540	Drafters	62,7
17-3013	1540	Drafters	62,7
17-3019	1540	Drafters	62,7
17-3022	1550	Engineering technicians, except drafters	57,4
17-3023	1550	Engineering technicians, except drafters	57,4
17-3025	1550	Engineering technicians, except drafters	57,4
17-3027	1550	Engineering technicians, except drafters	57,4
17-3029	1550	Engineering technicians, except drafters	57,4
17-3031	1560	Surveying and mapping technicians	49,6
19-1011	1600	Agricultural and food scientists	77,7
19-1012	1600	Agricultural and food scientists	77,7
19-1013	1600	Agricultural and food scientists	77,7
19-1021	1610	Biological scientists	85,8
19-1022	1610	Biological scientists	85,8
19-1023	1610	Biological scientists	85,8
19-1029	1610	Biological scientists	85,8
19-1031	1640	Conservation scientists and foresters	80,1
19-1041	1650	Medical scientists	88,0
19-1042	1650	Medical scientists	88,0
19-1099	1660	Life scientists, all other	88,0
19-2021	1710	Atmospheric and space scientists	85,9
19-2031	1720	Chemists and materials scientists	86,6
19-2032	1720	Chemists and materials scientists	86,6
19-2041	1740	Environmental scientists and geoscientists	86,7
19-2042	1740	Environmental scientists and geoscientists	86,7
19-2099	1760	Physical scientists, all other	86,8
19-3011	1800	Economists	91,9
19-3021	1800	Economists	91,9
19-3022	1815	Survey researchers	82,7
19-3031	1820	Psychologists	88,7
19-3039	1820	Psychologists	88,7
19-3041	1830	Sociologists	82,7
19-3051	1840	Urban and regional planners	88,7
19-3094	1860	Political scientists	82,7
19-3099	1860	Miscellaneous social scientists and related workers	82,7
19-4011	1900	Agricultural and food science technicians	44,5
19-4021	1910	Biological technicians	54,1
19-4031	1920	Chemical technicians	53,8
19-4041	1930	Geological and petroleum technicians	63,2
19-4061	1950	Social science research assistants	55,1
19-4091	1965	Environmental Science	55,1
19-4099	1965	Miscellaneous life, physical, and social science technicians	55,1

21-1011	2000	Counselors	63,7
21-1012	2000	Counselors	63,7
21-1013	2000	Counselors	63,7
21-1014	2000	Counselors	63,7
21-1015	2000	Counselors	63,7
21-1019	2000	Counselors	63,7
21-1021	2010	Social workers	62,9
21-1022	2010	Social workers	62,9
21-1023	2010	Social workers	62,9
21-1029	2010	Social workers	62,9
21-1092	2015	Probation officers and correctional treatment specialists	65,3
21-1093	2016	Social and human service assistants	43,8
21-1091	2025	Health educators and community health workers	53,8
21-1099	2025	Community and social service specialists, including health educatc	53,8
21-2011	2040	Clergy	62,6
21-2021	2050	Directors, religious activities and education	57,8
21-2099	2060	Religious workers, all other	51,7
23-1011	2100	Lawyers	91,1
23-1021	2110	Judges, magistrates, and other judicial workers	91,1
23-2011	2145	Paralegals and legal assistants	60,3
23-2091	2110	Judges, magistrates, and other judicial workers	91,1
23-2092	2105	Judicial law clerks	79,6
23-2093	2110	Judges, magistrates, and other judicial workers	91,1
23-2099	2160	Miscellaneous legal support workers	56,8
25-1011	2200	Postsecondary teachers	84,5
25-1021	2200	Postsecondary teachers	84,5
25-1022	2200	Postsecondary teachers	84,5
25-1032	2200	Postsecondary teachers	84,5
25-1041	2200	Postsecondary teachers	84,5
25-1042	2200	Postsecondary teachers	84,5
25-1052	2200	Postsecondary teachers	84,5
25-1054	2200	Postsecondary teachers	84,5
25-1061	2200	Postsecondary teachers	84,5
25-1063	2200	Postsecondary teachers	84,5
25-1065	2200	Postsecondary teachers	84,5
25-1066	2200	Postsecondary teachers	84,5
25-1067	2200	Postsecondary teachers	84,5
25-1069	2200	Postsecondary teachers	84,5
25-1071	2200	Postsecondary teachers	84,5
25-1072	2200	Postsecondary teachers	84,5
25-1081	2200	Postsecondary teachers	84,5
25-1111	2200	Postsecondary teachers	84,5
25-1112	2200	Postsecondary teachers	84,5
25-1113	2200	Postsecondary teachers	84,5
25-1121	2200	Postsecondary teachers	84,5
25-1122	2200	Postsecondary teachers	84,5
25-1123	2200	Postsecondary teachers	84,5
25-1124	2200	Postsecondary teachers	84,5

25-1125	2200	Postsecondary teachers	84,5
25-1126	2200	Postsecondary teachers	84,5
25-1191	2200	Postsecondary teachers	84,5
25-1199	2200	Postsecondary teachers	84,5
25-2011	2300	Preschool and kindergarten teachers	41,1
25-2012	2300	Preschool and kindergarten teachers	41,1
25-2021	2310	Elementary and middle school teachers	80,9
25-2022	2310	Elementary and middle school teachers	80,9
25-2023	2310	Elementary and middle school teachers	80,9
25-2031	2320	Secondary school teachers	82,5
25-2032	2320	Secondary school teachers	82,5
25-2041	2330	Special education teachers	71,7
25-2042	2330	Special education teachers	71,7
25-2043	2330	Special education teachers	71,7
25-3011	2340	Other teachers and instructors	59,1
25-3021	2340	Other teachers and instructors	59,1
25-3099	2340	Other teachers and instructors	59,1
25-4011	2400	Archivists, curators, and museum technicians	70,4
25-4021	2430	Librarians	77,2
25-4031	2440	Library technicians	34,6
25-9011	2550	Other education, training, and library workers	70,3
25-9021	2550	Other education, training, and library workers	70,3
25-9031	2550	Other education, training, and library workers	70,3
25-9041	2540	Teacher assistants	26,1
25-9099	2550	Other education, training, and library workers	70,3
27-1011	2600	Artists and related workers	60,1
27-1012	2600	Artists and related workers	60,1
27-1013	2600	Artists and related workers	60,1
27-1014	2600	Artists and related workers	60,1
27-1019	2600	Artists and related workers	60,1
27-1021	2630	Designers	61,4
27-1022	2630	Designers	61,4
27-1023	2630	Designers	61,4
27-1024	2630	Designers	61,4
27-1025	2630	Designers	61,4
27-1026	2630	Designers	61,4
27-1027	2630	Designers	61,4
27-1029	2630	Designers	61,4
27-2011	2700	Actors	55,0
27-2012	2710	Producers and directors	72,7
27-2021	2720	Athletes, coaches, umpires, and related workers	59,4
27-2022	2720	Athletes, coaches, umpires, and related workers	59,4
27-2023	2720	Athletes, coaches, umpires, and related workers	59,4
27-2031	2740	Dancers and choreographers	37,2
27-2032	2740	Dancers and choreographers	37,2
27-2041	2750	Musicians, singers, and related workers	52,3
27-2042	2750	Musicians, singers, and related workers	52,3
27-2099	2760	Entertainers and performers, sports and related workers, all other	49,3

27-3011	2800	Announcers	53,7
27-3012	2800	Announcers	53,7
27-3021	2810	News analysts, reporters and correspondents	72,5
27-3022	2810	News analysts, reporters and correspondents	72,5
27-3031	2825	Public relations specialists	74,6
27-3041	2830	Editors	73,4
27-3042	2840	Technical writers	77,0
27-3043	2850	Writers and authors	73,6
27-3099	2860	Miscellaneous media and communication workers	54,6
27-4013	2900	Broadcast and sound engineering technicians and radio operators	57,3
27-4014	2900	Broadcast and sound engineering technicians and radio operators	57,3
27-4021	2910	Photographers	51,5
27-4031	2920	Television, video, and motion picture camera operators and editor	62,6
27-4032	2920	Television, video, and motion picture camera operators and editor	62,6
27-4099	2960	Media and communication equipment workers, all other	57,3
29-1011	3000	Chiropractors	83,4
29-1021	3010	Dentists	92,1
29-1029	3010	Dentists	92,1
29-1031	3030	Dietitians and nutritionists	61,4
29-1041	3040	Optometrists	91,6
29-1051	3050	Pharmacists	92,0
29-1061	3060	Physicians and surgeons	92,8
29-1062	3060	Physicians and surgeons	92,8
29-1063	3060	Physicians and surgeons	92,8
29-1064	3060	Physicians and surgeons	92,8
29-1065	3060	Physicians and surgeons	92,8
29-1066	3060	Physicians and surgeons	92,8
29-1067	3060	Physicians and surgeons	92,8
29-1069	3060	Physicians and surgeons	92,8
29-1071	3110	Physician assistants	75,7
29-1081	3120	Podiatrists	88,3
29-1111	3255	Registered nurses	84,2
29-1122	3150	Occupational therapists	89,4
29-1123	3160	Physical therapists	87,3
29-1124	3200	Radiation therapists	86,0
29-1126	3220	Respiratory therapists	79,9
29-1127	3230	Speech-language pathologists	88,3
29-1129	3245	Therapists, all other	71,4
29-1131	3250	Veterinarians	89,9
29-1199	3260	Health diagnosing and treating practitioners, all other	69,5
29-2011	3300	Clinical laboratory technologists and technicians	60,5
29-2012	3300	Clinical laboratory technologists and technicians	60,5
29-2021	3310	Dental hygienists	76,7
29-2031	3420	Health practitioner support technologists and technicians	37,2
29-2032	3320	Diagnostic related technologists and technicians	69,3
29-2034	3420	Health practitioner support technologists and technicians	37,2
29-2041	3400	Emergency medical technicians and paramedics	53,0
29-2051	3420	Health practitioner support technologists and technicians	37,2

29-2052	3420	Health practitioner support technologists and technicians	37,2
29-2053	3420	Health practitioner support technologists and technicians	37,2
29-2055	3420	Health practitioner support technologists and technicians	37,2
29-2056	3420	Health practitioner support technologists and technicians	37,2
29-2061	3500	Licensed practical and licensed vocational nurses	46,6
29-2071	3510	Medical records and health information technicians	36,9
29-2081	3520	Opticians, dispensing	41,4
29-2091	3535	Miscellaneous health technologists and technicians	49,2
29-2099	3535	Miscellaneous health technologists and technicians	49,2
29-9011	3540	Other healthcare practitioners and technical occupations	64,2
29-9012	3540	Other healthcare practitioners and technical occupations	64,2
29-9091	3540	Other healthcare practitioners and technical occupations	64,2
29-9099	3540	Other healthcare practitioners and technical occupations	64,2
31-1011	3600	Nursing, psychiatric, and home health aides	24,2
31-1012	3600	Nursing, psychiatric, and home health aides	24,2
31-1013	3600	Nursing, psychiatric, and home health aides	24,2
31-2011	3610	Occupational therapy assistants and aides	71,1
31-2012	3610	Occupational therapy assistants and aides	71,1
31-2021	3620	Physical therapist assistants and aides	58,6
31-2022	3620	Physical therapist assistants and aides	58,6
31-9011	3630	Massage therapists	37,8
31-9091	3640	Dental assistants	28,6
31-9092	3645	Medical assistants	28,7
31-9093	3655	Miscellaneous healthcare support occupations, including medical	28,6
31-9094	3646	Medical transcriptionists	37,9
31-9095	3647	Pharmacy aides	35,5
39-9096	3648	Veterinary assistants and laboratory animal caretakers	25,6
31-9099	3655	Miscellaneous healthcare support occupations, including medical	28,6
33-1011	3700	First-line supervisors of correctional officers	54,3
33-1012	3710	First-line supervisors of police and detectives	72,6
33-1021	3720	First-line supervisors of fire fighting and prevention workers	75,2
33-1099	3730	First-line supervisors of protective service workers, all other	52,4
33-2011	3740	Firefighters	65,1
33-2021	3750	Fire inspectors	59,0
33-2022	3750	Fire inspectors	59,0
33-3011	3800	Bailiffs, correctional officers, and jailers	45,1
33-3012	3800	Bailiffs, correctional officers, and jailers	45,1
33-3021	3820	Detectives and criminal investigators	73,5
33-3031	3830	Fish and game wardens	50,1
33-3041	3840	Parking enforcement workers	50,1
33-3051	3850	Police and sheriff's patrol officers	65,3
33-3052	3860	Transit and railroad police	65,3
33-9011	3900	Animal control workers	32,7
33-9021	3910	Private detectives and investigators	63,3
33-9031	3930	Security guards and gaming surveillance officers	34,7
33-9032	3930	Security guards and gaming surveillance officers	34,7
33-9092	3955	Lifeguards and other recreational, and all other protective service v	36,8
33-9099	3955	Protective service workers	36,8

35-1011	4000	Chefs and head cooks	32,6
35-1012	4010	First-line supervisors of food preparation and serving workers	27,0
35-2011	4020	Cooks	13,6
35-2012	4020	Cooks	13,6
35-2013	4020	Cooks	13,6
35-2014	4020	Cooks	13,6
35-2015	4020	Cooks	13,6
35-2019	4020	Cooks	13,6
35-2021	4030	Food preparation workers	13,6
35-3011	4040	Bartenders	26,9
35-3021	4050	Combined food preparation and serving workers, including fast foc	14,0
35-3022	4060	Counter attendants, cafeteria, food concession, and coffee shop	14,6
35-3031	4110	Waiters and waitresses	19,6
35-3041	4120	Food servers, nonrestaurant	17,0
35-9011	4130	Dining room and cafeteria attendants and bartender helpers	13,3
35-9021	4140	Dishwashers	10,6
35-9031	4150	Hosts and hostesses, restaurant, lounge, and coffee shop	22,4
35-9099	4160	Food preparation and serving related workers, all other	13,3
37-1011	4200	First-line supervisors of housekeeping and janitorial workers	33,2
37-1012	4210	First-line supervisors of landscaping, lawn service, and groundskee	37,6
37-2011	4220	Janitors and building cleaners	20,7
37-2012	4230	Maids and housekeeping cleaners	12,6
37-2019	4220	Janitors and building cleaners	20,7
37-2021	4240	Pest control workers	32,8
37-3011	4250	Grounds maintenance workers	18,6
37-3012	4250	Grounds maintenance workers	18,6
37-3013	4250	Grounds maintenance workers	18,6
37-3019	4250	Grounds maintenance workers	18,6
39-1011	4300	First-line supervisors of gaming workers	46,7
39-1021	4320	First-line supervisors of personal service workers	39,6
39-2011	4340	Animal trainers	37,4
39-2021	4350	Nonfarm animal caretakers	26,5
39-3011	4400	Gaming services workers	37,1
39-3031	4420	Ushers, lobby attendants, and ticket takers	25,4
39-3099	4430	Miscellaneous entertainment attendants and related workers	29,4
39-4021	4460	Embalmers and funeral attendants	41,2
39-5011	4500	Barbers	21,5
39-5012	4510	Hairdressers, hairstylists, and cosmetologists	24,6
39-5091	4520	Miscellaneous personal appearance workers	20,1
39-5092	4520	Miscellaneous personal appearance workers	20,1
39-5094	4520	Miscellaneous personal appearance workers	20,1
39-6011	4530	Baggage porters, bellhops, and concierges	29,5
39-6012	4530	Concierges	29,5
39-6031	9050	Flight attendants	52,6
39-6032	9415	Transportation attendants, except flight attendants	33,3
39-9011	4600	Childcare workers	21,8
39-9021	4610	Personal care aides	20,1
39-9031	4620	Recreation and fitness workers	42,9

39-9032	4620	Recreation and fitness workers	42,9
39-9041	4640	Residential advisors	29,3
39-9099	4650	Personal care and service workers, all other	27,6
41-1011	4700	First-line supervisors of retail sales workers	43,0
41-1012	4710	First-line supervisors of non-retail sales workers	57,5
41-2011	4720	Cashiers	21,6
41-2012	4720	Cashiers	21,6
41-2021	4740	Counter and rental clerks	30,9
41-2022	4750	Parts salespersons	29,6
41-2031	4760	Retail salespersons	39,7
41-3011	4800	Advertising sales agents	61,5
41-3021	4810	Insurance sales agents	57,8
41-3031	4820	Securities, commodities, and financial services sales agents	71,0
41-3041	4830	Travel agents	45,0
41-3099	4840	Sales representatives, services, all other	61,2
41-4011	4850	Sales representatives, wholesale and manufacturing	62,0
41-4012	4850	Sales representatives, wholesale and manufacturing	62,0
41-9011	4900	Models, demonstrators, and product promoters	38,9
41-9021	4920	Real estate brokers and sales agents	56,9
41-9022	4920	Real estate brokers and sales agents	56,9
41-9031	4930	Sales engineers	86,5
41-9041	4940	Telemarketers	30,3
41-9091	4950	Door-to-door sales workers, news and street vendors, and related w	32,6
41-9099	4965	Sales and related workers, all other	55,3
43-1011	5000	First-line supervisors of office and administrative support workers	52,0
43-2011	5010	Switchboard operators, including answering service	26,8
43-2021	5020	Telephone operators	31,9
43-2099	5030	Communications equipment operators, all other	48,1
43-3011	5100	Bill and account collectors	34,8
43-3021	5110	Billing and posting clerks	33,4
43-3031	5120	Bookkeeping, accounting, and auditing clerks	38,3
43-3051	5140	Payroll and timekeeping clerks	41,8
43-3061	5150	Procurement clerks	53,0
43-3071	5160	Tellers	20,8
43-4011	5200	Brokerage clerks	57,1
43-4021	5210	Correspondence clerks	32,0
43-4031	5220	Court, municipal, and license clerks	41,1
43-4041	5230	Credit authorizers, checkers, and clerks	39,5
43-4051	5240	Customer service representatives	37,6
43-4061	5250	Eligibility interviewers, government programs	52,9
43-4071	5260	File Clerks	35,3
43-4081	5300	Hotel, motel, and resort desk clerks	24,1
43-4121	5320	Library assistants, clerical	36,5
43-4131	5330	Loan interviewers and clerks	42,7
43-4141	5340	New accounts clerks	39,2
43-4151	5350	Order clerks	32,0
43-4161	5360	Human resources assistants, except payroll and timekeeping	44,6
43-4171	5400	Receptionists and information clerks	25,7

43-4181	5410	Reservation and transportation ticket agents and travel clerks	44,9
43-4199	5420	Information and record clerks, all other	43,1
43-5011	5500	Cargo and freight agents	40,1
43-5021	5510	Couriers and messengers	39,9
43-5031	5520	Dispatchers	38,2
43-5032	5520	Dispatchers	38,2
43-5041	5530	Meter readers, utilities	37,0
43-5051	5540	Postal service clerks	59,8
43-5052	5550	Postal service mail carriers	58,4
43-5053	5560	Postal service mail sorters, processors, and processing machine ope	58,2
43-5061	5600	Production, planning, and expediting clerks	49,4
43-5071	5610	Shipping, receiving, and traffic clerks	23,7
43-5081	5620	Stock clerks and order fillers	23,8
43-5111	5630	Weighers, measurers, checkers, and samplers, recordkeeping	31,6
43-6011	5700	Secretaries and administrative assistants	38,8
43-6012	5700	Secretaries and administrative assistants	38,8
43-6013	5700	Secretaries and administrative assistants	38,8
43-6014	5700	Secretaries and administrative assistants	38,8
43-9011	5800	Computer operators	47,9
43-9021	5810	Data entry keyers	32,6
43-9022	5820	Word processors and typists	35,1
43-9031	5830	Desktop publishers	45,3
43-9041	5840	Insurance claims and policy processing clerks	39,2
43-9051	5850	Mail clerks and mail machine operators, except postal service	25,7
43-9061	5860	Office clerks, general	35,8
43-9071	5900	Office machine operators, except computer	29,2
43-9081	5910	Proofreaders and copy markers	50,1
43-9111	5920	Statistical assistants	49,6
43-9199	5940	Office and administrative support workers, all other	45,3
45-1011	6005	First-line supervisors of farming, fishing, and forestry workers	34,7
45-1012	6010	Agricultural inspectors	51,6
45-2011	6010	Agricultural inspectors	51,6
45-2021	6020	Animal breeders	14,0
45-2041	6040	Graders and sorters, agricultural products	14,0
45-2091	6050	Miscellaneous agricultural workers	14,0
45-2092	6050	Miscellaneous agricultural workers	14,0
45-2093	6050	Miscellaneous agricultural workers	14,0
45-2099	6050	Miscellaneous agricultural workers	14,0
45-4011	6120	Forest and conservation workers	31,5
45-4021	6130	Logging workers	20,0
45-4022	6130	Logging workers	20,0
45-4029	6130	Logging workers	20,0
47-1011	6200	First-line supervisors of construction trades and extraction workers	46,0
47-2011	6210	Boilermakers	42,2
47-2021	6220	Brickmasons, blockmasons, and stonemasons	24,6
47-2022	6220	Brickmasons, blockmasons, and stonemasons	24,6
47-2031	6230	Carpenters	28,7
47-2041	6240	Carpet, floor, and tile installers and finishers	23,0

47-2042	6240	Carpet, floor, and tile installers and finishers	23,0
47-2043	6240	Carpet, floor, and tile installers and finishers	23,0
47-2044	6240	Carpet, floor, and tile installers and finishers	23,0
47-2051	6250	Cement masons, concrete finishers, and terrazzo workers	21,4
47-2061	6260	Construction laborers	25,2
47-2071	6300	Paving, surfacing, and tamping equipment operators	22,9
47-2072	6310	Pile-driver operators	31,0
47-2073	6320	Operating engineers and other construction equipment operators	31,0
47-2081	6330	Drywall installers, ceiling tile installers, and tapers	19,2
47-2082	6330	Tapers	19,2
47-2111	6355	Electricians	45,4
47-2131	6400	Insulation workers	27,6
47-2132	6400	Insulation workers	27,6
47-2141	6420	Painters, construction and maintenance	24,0
47-2151	6440	Pipelayers	36,2
47-2152	6440	Plumbers, pipefitters, and steamfitters	36,2
47-2161	6460	Plasterers and stucco masons	20,9
47-2171	6500	Reinforcing iron and rebar workers	25,6
47-2181	6515	Roofers	20,5
47-2211	6520	Sheet metal workers	34,1
47-2221	6530	Structural iron and steel workers	35,2
47-3011	6600	Helpers, construction trades	19,7
47-3012	6600	Helpers, construction trades	19,7
47-3013	6600	Helpers, construction trades	19,7
47-3014	6430	Paperhangers	36,8
47-3015	6600	Helpers, construction trades	19,7
47-3016	6600	Helpers, construction trades	19,7
47-3019	6600	Helpers, construction trades	19,7
47-4011	6660	Construction and building inspectors	53,9
47-4021	6700	Elevator installers and repairers	57,1
47-4031	6710	Fence erectors	23,8
47-4041	6720	Hazardous materials removal workers	34,8
47-4051	6730	Highway maintenance workers	27,2
47-4061	6740	Rail-track laying and maintenance equipment operators	40,0
47-4071	6750	Septic tank servicers and sewer pipe cleaners	31,4
47-4091	6765	Miscellaneous construction and related workers	31,4
47-4099	6765	Miscellaneous construction and related workers	31,4
47-5011	6800	Derrick, rotary drill, and service unit operators, oil, gas, and mining	36,4
47-5012	6800	Derrick, rotary drill, and service unit operators, oil, gas, and mining	36,4
47-5013	6800	Derrick, rotary drill, and service unit operators, oil, gas, and mining	36,4
47-5021	6820	Earth drillers, except oil and gas	35,8
47-5031	6830	Explosives workers, ordnance handling experts, and blasters	41,8
47-5041	6840	Mining machine operators	42,2
47-5049	6840	Mining machine operators	42,2
47-5051	6910	Roof bolters, mining	36,7
47-5061	6910	Roof bolters, mining	36,7
47-5071	6920	Roustabouts, oil and gas	36,4
47-5081	6930	Helpers--extraction workers	36,7

47-5099	6940	Other extraction workers	36,7
49-1011	7000	First-line supervisors of mechanics, installers, and repairers	52,4
49-2011	7010	Computer, automated teller, and office machine repairers	51,8
49-2021	7020	Radio and telecommunications equipment installers and repairers	52,1
49-2022	7020	Radio and telecommunications equipment installers and repairers	52,1
49-2091	7030	Avionics technicians	53,1
49-2092	7040	Electric motor, power tool, and related repairers	39,6
49-2093	7050	Electrical and electronics installers and repairers, transportation e	53,5
49-2094	7100	Electrical and electronics repairers, industrial and utility	53,5
49-2095	7100	Electrical and electronics repairers, industrial and utility	53,5
49-2096	7110	Electronic equipment installers and repairers, motor vehicles	39,0
49-2097	7120	Electronic home entertainment equipment installers and repairers	38,1
49-2098	7130	Security and fire alarm systems installers	42,9
49-3011	7140	Aircraft mechanics and service technicians	53,6
49-3021	7150	Automotive body and related repairers	28,3
49-3022	7160	Automotive glass installers and repairers	23,8
49-3023	7200	Automotive service technicians and mechanics	31,2
49-3031	7210	Bus and truck mechanics and diesel engine specialists	35,3
49-3041	7220	Heavy vehicle and mobile equipment service technicians and mech	39,2
49-3042	7220	Heavy vehicle and mobile equipment service technicians and mech	39,2
49-3043	7260	Miscellaneous vehicle and mobile equipment mechanics, installers	21,3
49-3053	7240	Small engine mechanics	27,4
49-3092	7260	Miscellaneous vehicle and mobile equipment mechanics, installers	21,3
49-3093	7260	Miscellaneous vehicle and mobile equipment mechanics, installers	21,3
49-9012	7300	Control and valve installers and repairers	41,6
49-9021	7315	Heating, air conditioning, and refrigeration mechanics and installe	38,8
49-9031	7320	Home appliance repairers	33,9
49-9041	7330	Industrial and refractory machinery mechanics	42,8
49-9042	7340	Maintenance and repair workers, general	36,4
49-9043	7350	Maintenance workers, machinery	37,3
49-9045	7330	Industrial and refractory machinery mechanics	42,8
49-9051	7410	Electrical power-line installers and repairers	51,9
49-9052	7420	Telecommunications line installers and repairers	46,2
49-9063	7430	Precision instrument and equipment repairers	53,9
49-9069	7430	Precision instrument and equipment repairers	53,9
49-9091	7510	Coin, vending, and amusement machine servicers and repairers	30,3
49-9092	7520	Commercial divers	35,4
49-9093	7540	Locksmiths and safe repairers	35,1
49-9095	7550	Manufactured building and mobile home installers	21,2
49-9096	7560	Riggers	42,3
49-9097	7600	Signal and track switch repairers	35,4
49-9098	7610	Helpers--installation, maintenance, and repair workers	22,9
49-9099	7630	Other installation, maintenance, and repair workers	35,4
51-1011	7700	First-line supervisors of production and operating workers	46,5
51-2011	7710	Aircraft structure, surfaces, rigging, and systems assemblers	37,7
51-2021	7710	Aircraft structure, surfaces, rigging, and systems assemblers	37,7
51-2022	7720	Electrical, electronics, and electromechanical assemblers	23,3
51-2023	7720	Electrical, electronics, and electromechanical assemblers	23,3

51-2031	7730	Engine and other machine assemblers	34,4
51-2041	7740	Structural metal fabricators and fitters	33,9
51-2091	7750	Miscellaneous assemblers and fabricators	25,0
51-2092	7750	Miscellaneous assemblers and fabricators	25,0
51-2093	7750	Miscellaneous assemblers and fabricators	25,0
51-2099	7750	Miscellaneous assemblers and fabricators	25,0
51-3011	7800	Bakers	19,7
51-3021	7810	Butchers and other meat, poultry, and fish processing workers	18,8
51-3022	7810	Butchers and other meat, poultry, and fish processing workers	18,8
51-3023	7855	Food processing workers, all other	21,6
51-3091	7830	Food and tobacco roasting, baking, and drying machine operators a	27,4
51-3092	7840	Food batchmakers	20,5
51-3093	7850	Food cooking machine operators and tenders	18,9
51-4011	7900	Computer control programmers and operators	38,4
51-4012	7900	Computer control programmers and operators	38,4
51-4021	7920	Extruding and drawing machine setters, operators, and tenders, me	28,1
51-4022	7930	Forging machine setters, operators, and tenders, metal and plastic	28,3
51-4031	7950	Cutting, punching, and press machine setters, operators, and tende	22,9
51-4033	8000	Grinding, lapping, polishing, and buffing machine tool setters, ope	23,2
51-4034	8010	Lathe and turning machine tool setters, operators, and tenders, me	27,5
51-4035	8020	Milling and planing machine setters, operators, and tenders, metal	25,1
51-4041	8030	Machinists	38,3
51-4051	8040	Metal furnace operators, tenders, pourers, and casters	33,7
51-4052	8040	Metal furnace operators, tenders, pourers, and casters	33,7
54-4071	8060	Model makers and patternmakers, metal and plastic	51,8
51-4072	8100	Molders and molding machine setters, operators, and tenders, met	26,1
51-4081	8120	Multiple machine tool setters, operators, and tenders, metal and p	25,1
51-4111	8130	Tool and die makers	48,3
51-4121	8140	Welding, soldering, and brazing workers	28,4
51-4122	8140	Welding, soldering, and brazing workers	28,4
51-4191	8150	Heat treating equipment setters, operators, and tenders, metal and	32,1
51-4193	8200	Plating and coating machine setters, operators, and tenders, metal	27,7
51-4194	8210	Tool grinders, filers, and sharpeners	25,5
51-4199	8220	Metal workers and plastic workers, all other	25,1
51-5011	8256	Print binding and finishing workers	23,3
51-5021	8256	Print binding and finishing workers	23,3
51-5022	8250	Prepress technicians and workers	34,6
51-5023	8255	Printing press operators	31,1
51-6011	8300	Laundry and dry-cleaning workers	15,8
51-6021	8310	Pressers, textile, garment, and related materials	12,4
51-6031	8320	Sewing machine operators	13,2
51-6062	8400	Textile cutting machine setters, operators, and tenders	18,8
51-6063	8410	Textile knitting and weaving machine setters, operators, and tende	12,7
51-6064	8420	Textile winding, twisting, and drawing out machine setters, operat	11,8
51-6091	8430	Extruding and forming machine setters, operators, and tenders, syr	29,9
51-6092	8440	Fabric and apparel patternmakers	29,9
51-6099	8460	Textile, apparel, and furnishings workers, all other	29,9
51-7011	8500	Cabinetmakers and bench carpenters	27,1

51-7021	8510	Furniture finishers	24,6
51-7031	8520	Model makers and patternmakers, wood	28,8
51-7041	8530	Sawing machine setters, operators, and tenders, wood	14,4
51-7042	8540	Woodworking machine setters, operators, and tenders, except saw	17,1
51-7099	8550	Woodworkers, all other	28,8
51-8012	8600	Power plant operators, distributors, and dispatchers	65,2
51-8013	8600	Power plant operators, distributors, and dispatchers	65,2
51-8021	8610	Stationary engineers and boiler operators	50,0
51-8031	8620	Water and wastewater treatment plant and system operators	43,5
51-8091	8640	Chemical processing machine setters, operators, and tenders	48,8
51-8092	8640	Chemical processing machine setters, operators, and tenders	48,8
51-8093	8640	Chemical processing machine setters, operators, and tenders	48,8
51-8099	8630	Miscellaneous plant and system operators	53,2
51-9011	8640	Chemical processing machine setters, operators, and tenders	48,8
51-9012	8650	Crushing, grinding, polishing, mixing, and blending workers	27,7
51-9021	8650	Crushing, grinding, polishing, mixing, and blending workers	27,7
51-9023	8650	Crushing, grinding, polishing, mixing, and blending workers	27,7
51-9031	8710	Cutting workers	18,6
51-9032	8710	Cutting workers	18,6
51-9041	8720	Extruding, forming, pressing, and compacting machine setters, ope	24,8
51-9051	8730	Furnace, kiln, oven, drier, and kettle operators and tenders	39,8
51-9061	8740	Inspectors, testers, sorters, samplers, and weighers	38,1
51-9071	8750	Jewelers and precious stone and metal workers	34,6
51-9082	8760	Medical, dental, and ophthalmic laboratory technicians	37,2
51-9111	8800	Packaging and filling machine operators and tenders	17,1
51-9121	8810	Painting workers	26,2
51-9122	8810	Painting workers	26,2
51-9123	8810	Painting workers	26,2
51-9131	8830	Photographic process workers and processing machine operators	34,5
51-9132	8830	Photographic process workers and processing machine operators	34,5
51-9141	8840	Semiconductor processors	26,8
51-9191	8850	Adhesive bonding machine operators and tenders	22,5
51-9193	8900	Cooling and freezing equipment operators and tenders	26,8
51-9194	8910	Etchers and engravers	29,5
51-9195	8920	Molders, shapers, and casters, except metal and plastic	31,9
51-9196	8930	Paper goods machine setters, operators, and tenders	32,7
51-9197	8940	Tire builders	36,4
51-9198	8950	Helpers--production workers	20,8
51-9199	8965	Production workers, all other	43,9
53-1011	9000	Supervisors of transportation and material moving workers	46,6
53-1021	9000	Supervisors of transportation and material moving workers	46,6
53-1031	9000	Supervisors of transportation and material moving workers	46,6
53-2011	9030	Aircraft pilots and flight engineers	79,4
53-2021	9040	Air traffic controllers and airfield operations specialists	66,4
53-2022	9040	Air traffic controllers and airfield operations specialists	66,4
53-3011	9110	Ambulance drivers and attendants, except emergency medical tech	26,7
53-3021	9120	Bus drivers	31,6
53-3022	9120	Bus drivers	31,6

53-3031	9130	Driver/sales workers and truck drivers	32,0
53-3032	9130	Driver/sales workers and truck drivers	32,0
53-3033	9130	Driver/sales workers and truck drivers	32,0
53-3041	9140	Taxi drivers and chauffeurs	28,5
53-3099	9150	Motor vehicle operators, all other	24,0
53-4011	9200	Locomotive engineers and operators	60,4
53-4021	9230	Railroad brake, signal, and switch operators	50,0
53-4031	9240	Railroad conductors and yardmasters	58,1
53-4099	9260	Subway, streetcar, and other rail transportation workers	48,4
53-5011	9300	Sailors and marine oilers	40,1
53-5021	9310	Ship and boat captains and operators	52,3
53-5031	9330	Ship engineers	40,1
53-6021	9350	Parking lot attendants	22,1
53-6031	9360	Automotive and watercraft service attendants	21,0
53-6041	9360	Automotive and watercraft service attendants	21,0
53-6051	9410	Transportation inspectors	50,1
53-6099	9420	Other transportation workers	41,6
53-7011	9500	Conveyor operators and tenders	33,1
53-7021	9510	Crane and tower operators	38,8
53-7032	9520	Dredge, excavating, and loading machine operators	30,1
53-7033	9520	Dredge, excavating, and loading machine operators	30,1
53-7041	9560	Hoist and winch operators	33,1
53-7051	9600	Industrial truck and tractor operators	20,9
53-7062	9620	Laborers and freight, stock, and material movers, hand	23,3
53-7063	9630	Machine feeders and offbearers	21,1
53-7064	9640	Packers and packagers, hand	14,8
53-7072	9650	Pumping station operators	43,4
53-7081	9720	Refuse and recyclable material collectors	24,1
53-7121	9740	Tank car, truck, and ship loaders	31,3
53-7199	9750	Material moving workers, all other	31,3
55-1011	9800	Military officer special and tactical operations leaders	70,1
55-1012	9800	Military officer special and tactical operations leaders	70,1
55-1015	9800	Military officer special and tactical operations leaders	70,1
55-1017	9800	Military officer special and tactical operations leaders	70,1
55-1019	9800	Military officer special and tactical operations leaders	70,1
55-2012	9810	First-line enlisted military supervisors	63,1
55-2013	9810	First-line enlisted military supervisors	63,1
55-3011	9820	Military enlisted tactical operations and air/weapons specialists an	39,1
55-3012	9830	Military, rank not specified	50,4
55-3015	9830	Military, rank not specified	50,4
55-3016	9830	Military, rank not specified	50,4
55-3018	9830	Military, rank not specified	50,4
55-3019	9820	Military enlisted tactical operations and air/weapons specialists an	39,1
