# Supplementary material for: Socioeconomic status across the early life course predicts gene expression signatures of disease and senescence
Source: J Epidemiol Community Health. 2024 Aug 29;78(12):e221812. doi: 10.1136/jech-2023-221812 (PMC11671950; doi:10.1136/jech-2023-221812)
Supplement: online supplemental file 3 [file jech-78-12-s003.pdf]

Supplementary Information

Socioeconomic Status Across the Early Life Course Predicts Gene Expression Signatures of Disease and Senescence

Cecilia Potente, Julien Bodelet, Hira Himeri, Steve Cole, Kathleen Harris, Michael Shanahan

Appendix 1
Details on construction of SES composite

Socioeconomic status composites at three different time points represent the sum of the standardized indicators of education, income and occupation.

ses_composite = (occupation + education + income)

Where occupation, education and income are quantified as described in the main text. The standardization of each of the socioeconomic indicators is done by normalizing the variable with respect to the mean and standard deviation using R's scale function.

Details on construction Socioeconomic Index (SEI)

- *Occupation SEI of parents (using information in Wave I and Wave II).*

The variables we used to create parents' SEI are:
H1RM4, H2RM4: resident mother occupation categories at Wave I and Wave II.
H1RF4, H2RF4: resident father occupation categories at Wave I and Wave II.

If occupation categories at Wave I is not available, we used occupation categories at Wave II. Parents SEI was created using the categorization suggested by Hauser and Warren (1997), where they have created the SEI for 1990 Census occupation. Wave I was collected in 1995 and Wave II in 1996.
The occupation categories in AddHealth are similar to the ones in Hauser and Warren (1997), but not always an exact match. Therefore, some occupation categories are taken approximately. The following table presents all the details.

Table S1- Details on construction of *parental SEI*

**Occupation categories in Hauser and Warren (1997) with the corresponding SEI scores in parenthesis**	**Occupation categories in our database in details**
Professional Specialty Occupations (60.92)	1: Professional, such as doctor, lawyer, scientist,<br>2: Professional, such as teacher, librarian, nurse
Executive, Administrative, and Managerial Occupations (40.22)	3: manager, such as executive, director
Technicians and Related Support Occupations (47.05)	4: technical, such as computer specialist, radiologist
Administrative Support Occupations, Including Clerical (32.24)	5: office worker, such as bookkeeper, office clerk, secretary

Sales Occupations (36.24)	6: sales worker, such as insurance agent, store clerk
Private Household Occupations (16.87)** Service Occupations, Except Protective and Household (21.96)**	7: restaurant worker or personal service, such as waitress, housekeeper
Precision Production, Craft, and Repair Occupations (31.51)	8: craftsperson, such as toolmaker, woodworker
	9: construction worker, such as carpenter, crane operator
	10: mechanic, such as electrician, plumber, machinist
Machine Operators, Assemblers, and Inspectors (22.58)	11: factory worker or laborer, such as assembler, janitor
Transportation and Material Moving Occupations (26.50)	12: transportation, such as bus driver, taxi driver
Protective Service Occupations (39.29)	military or security, such as police officer, soldier, fire fighter
Farming, Forestry, and Fishing Occupations (23.34)	farm or fishery worker
In order not to lose this part of respondents, I used all occupations average SEI (36.81)?	other

** We took the mean of these two categories SEI scores for category 7.

- *Occupation SEI in Wave IV*

The variable we used to create SEI in Wave IV is:
H4LM18

At Wave IV, the 2000 Standard Occupational Classification (SOC) system was used to classify Add Health respondents' first full-time job and their current/most recent paying job that was at least 10 hours per week, excluding military service. The SOC version used at Wave IV was created on December 4, 2001 by the Bureau of Labor Statistics', downloaded from their web site in 2007. We used Hout and colleagues prestige scores (2014). To be able to use the same source for prestige scores in Wave IV and Wave V we did the crosswalk between SOC2000 and SOC2010 (see online Dataset S1), since Hout and colleagues (2014) have created the prestige scores only for SOC2010 and not for SOC2000.

- *Occupation SEI in Wave V*

The variables we used to create SEI in Wave V are:
H5LM12, if H5LM12 is not available, then we used H5LM22 (respondents' past occupation code).

At Wave V, the 2010 Standard Occupational Classification (SOC) system was used to classify Add Health respondents' first full-time job and their current/most recent paying job. Respondent SEI at Wave V was created using the scheme developed by Hout (2014). They have created the corresponding SEI for the 2010 occupation codes. Therefore, we linked our respondents' 2010 occupation codes to SEI directly based on Hout (2014).

Appendix 2
Software packages

Normalization of the raw mRNA-seq counts is based on weighted trimmed mean of log expression ratios (TMM normalization) using the *edgeR* package in R (Robinson, McCarthy, and Smyth 2009). We also corrected for batch effects using the *ComBat* function in the *sva* package in R (Leek et al. 2012). We selected 13 disease and senescence signatures reflecting common chronic conditions in the American population and, for each signature, use Sparse Principal Component Analysis (SPCA) to reduce dimensionality using the *PMA* package in R (Tibshirani 2020); the optimal number of sparse principal components (PCs) was identified using *findPC* package in R (Zhuang, Wang, and Ji 2022). The direct and average causal mediated effects are estimated in a counterfactual framework using *brms* in R (Bürkner 2017).

Appendix 3

Rstan Model with Details of Imputation Variables

Let $k = 1, \dots, K$ denotes the index of the PCs over the 13 signatures.

For each PC y_k we have a RLM

$$y_k = \alpha_{0,k} + \delta_k \sum_{t=1}^{3} w_{t,k} x_t + \sum_{j=1}^{J} \alpha_{j,k} C_j + \varepsilon_k$$

where x_t denotes SES at time t, C_j are non-time varying covariates, and ε_k are random errors. The parameters δ_k reflect the global effect and $w_{t,k}$ the relative effect, which is such that $\sum_{t=1}^{3} w_{t,k} = 1$, $\alpha_{0,k}$is the intercept, and $\alpha_{j,k}$ the effect of the jth covariate. We consider the following prior distributions, for $k = 1, \dots, K$:

$$\begin{gathered}
(w_{1,k}, w_{2,k}, w_{3,k}) \sim Dir(1,1,1) \\
\varepsilon_k \sim N(0, \sigma_k^2), k = 1, \dots, K \\
\sigma_k \sim logN(1, 1) \\
\delta_k \sim (1 - \pi_k) N(\mu, \gamma) + \pi_k \Delta_0 \\
\pi_k \sim U(0,1) \\
\mu \sim N(0, 10) \\
\gamma \sim logN(1,1) \\
\alpha_{j,k} \sim N(0,1), j = 0,1, \dots, J
\end{gathered}$$

where Δ_0 denotes the Point mass distribution at 0 (or Dirac distribution). The prior distribution δ_k is then assumed to be a mixture between the Dirac. The mixture coefficient π is the probability that δ is 0 (i.e. with non-credible association). This modelling allows to take care of the multiple comparison issue. Moreover, since $x_{1,k}$ may contain missing values, we use a Bayesian imputation as follows

$$x_{1,k} \sim N(\lambda_0 + \sum_{l=1}^{L} \lambda_l u_l \, , \sigma_x)$$
$$\lambda_l \sim N(0, 1)$$
$$\sigma_x \sim logN(1, 1)$$

where u is a vector of covariates that predict $x_{1,k}$.

List of u predictors used for imputation of parental income from Wave I:

PA13	*Do you work outside the home?*
PA15	*Were you employed full time at your last job?*
PA16	*Are you unemployed right now, but looking for a job?*
PA17	*Are you employed full time?*
PA18	*Are you disabled?*
PA19	*Are you retired from a job?*
PA21	*Are you receiving public assistance, such as welfare?*
PA28C	*Please tell me whether each of the following statements is true with regard to your present neighborhood: You moved to this neighborhood because you had outgrown your previous housing.*
PA28D	*Please tell me whether each of the following statements is true with regard to your present neighborhood: You live in this neighborhood because you can afford better housing here than you could afford in other neighborhoods.*
PA28E	*Please tell me whether each of the following statements is true with regard to your present neighborhood: You live here because there is less crime in this neighborhood than there is in other neighborhoods.*
PA28F	*Please tell me whether each of the following statements is true with regard to your present neighborhood: You live here because there is less drug use and other illegal activity by adolescents in this neighborhood.*
PA28H	*Please tell me whether each of the following statements is true with regard to your present neighborhood: You live here because the schools here are better than they are in other neighborhoods.*
PA33	*In this neighborhood, how big a problem is litter or trash on the streets and sidewalks?*
PA34	*In this neighborhood, how big a problem are drug dealers and drug users?*
PA55	*About how much total income, before taxes did your family receive in 1994? Include your own income, the income of everyone else in your household, and income from welfare benefits, dividends, and all other sources.*
PA56	*Do you have enough money to pay your bills?*
PA57A	*Last month, did you or any member of your household receive: Social Security or Railroad Retirement?*
PA57B	*Last month, did you or any member of your household receive: Supplemental Security Income (SSI)?*
PA57C	*Last month, did you or any member of your household receive: Aid to Families with Dependent Children (AFDC)?*
PA57D	*Last month, did you or any member of your household receive: food stamps?*
PA57E	*Last month, did you or any member of your household receive: unemployment or workers compensations?*

Sensitivity to prior distribution

In order to validate the sensitivity to the prior distributions, we consider the following alternative priors for the main model

$$(w_{1,k}, w_{2,k}, w_{3,k}) \sim Dir(1,1,1)$$
$$\varepsilon_k \sim N(0, \sigma_k^2), k = 1, \dots, K$$
$$\sigma_k \sim logN(1, 1)$$
$$\delta_k \sim (1 - \pi_k)Cauchy(0,2.5) + \pi_k \Delta_0$$
$$\pi_k \sim U(0,1)$$
$$\alpha_{j,k} \sim Cauchy(0,2.5), j = 0,1, \dots, J$$

and for the imputation model:

$$x_{1,k} \sim N(\lambda_0 + \sum_{l=1}^{L} \lambda_l u_l \,, \sigma_x)$$
$$\lambda_l \sim Cauchy(0,2.5)$$
$$\sigma_x \sim logN(1, 1)$$

Results of this alternative specification are presented in Appedix 6.

Appendix 4
Table S2. Definition of mRNA-based disease signatures and validation

Outcomes	Validation	N. Genes	Type of study	Sources
Cardiovascular Disease (CVD)	95 variants (explaining 13.3 ± 0.4% of CAD heritability), 93 variants (explaining 12.9 ± 0.4% of CAD heritability), 109 variants (explaining a further 9.3 ± 0.3% of CAD heritability)	65	GWAS	(Nikpay et al. 2015)
Diabetes	19.6 % (SNP heritability)	139	GWAS	(Xue et al. 2018)
Inflammation	NA	1027	GWAS	(Loza et al. 2007)
Lupus	NA	171	gene expression in RNA from peripheral blood mononuclear cells (PBMC)	(Baechler et al. 2003)
Colorectal Cancer	Sensitivity, accuracy and specificity overall greater than 80 %	214	gene expression in RNA from peripheral blood mononuclear cells (PBMC)	(Guinney et al. 2015)

Outcomes	Validation	N. Genes	Type of study	Sources
Rheumatoid Arthritis	NA	43	gene expression in RNA from peripheral blood mononuclear cells (PBMC)	(Olsen et al. 2004)
Alzheimers	0.73 ROC AUC<br>chance that model will be able to distinguish between positive class and negative class.	170	gene expression in RNA from peripheral blood mononuclear cells (PBMC)	(Sood et al. 2015)
Asthma	NA	148	gene expression in RNA from peripheral blood mononuclear cells (PBMC)	(Alrashoudi et al. 2018)
Hypertension	Prediction of the left-out specimen was completed with 95% or 100% accuracy using different algorithms	106	gene expression in RNA from peripheral blood mononuclear cells (PBMC)	(Bull et al. 2004)
Aortic Aneurysm	Overall classification accuracy (average 78±6%), sensitivity (average 81±6%) and specificity (average 75±6%)	41	gene expression in RNA from peripheral blood mononuclear cells (PBMC)	(Wang et al. 2007)
Senescence	1,497 replicated age associated genes have been checked in other tissues and ethnicities ($p<0.05$)	1497	gene expression in RNA from peripheral blood mononuclear cells (PBMC)	(Peters et al. 2015)
COPD	Explained 20 % of the variation in FEV1 (without covariates) and<br>25 % (of the variation in FEV1 with covariates)	46	gene expression in RNA from peripheral blood mononuclear cells (PBMC)	(Bahr et al. 2013)

Appendix 5
Correlation matrix among SES Composites in the 3 time points

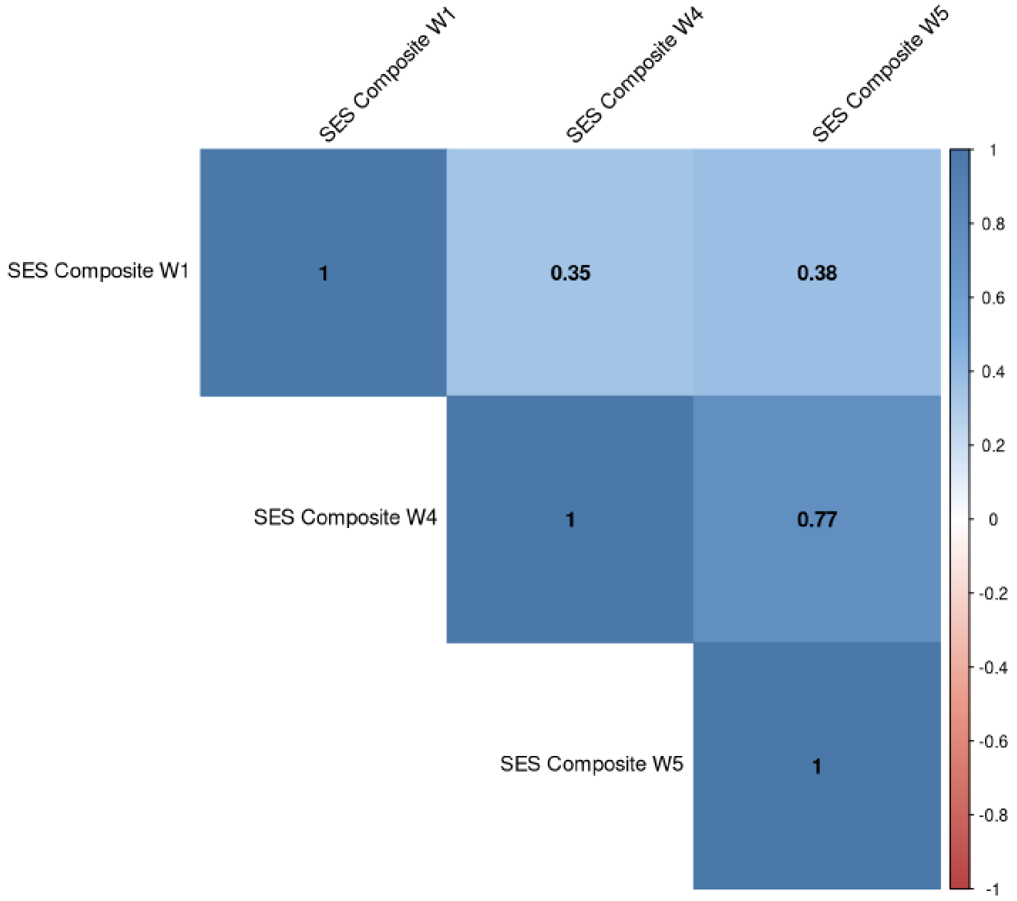

Appendix 6

Table S3 – Probability of regions of practical equivalence (ROPEs) for three broad life-course models based on Chumbley et al. (2021) including marital status as covariate.

Signatures	Accumulation	Sensitive	Critical
CVD (PC1)	0.01	0.80	0.22
Lupus (PC5)	0.008	0.66	0.33
Colorectal (PC3)	0.015	0.90	0.12
RA (PC1)	0.002	0.6	0.41
RA (PC4)	0.01	0.62	0.37
RA (PC5)	0.03	0.83	0.15
Asthma (PC2)	0.02	0.74	0.25
Asthma (PC3)	0.01	0.74	0.25
Diabetes (PC3)	0.005	0.63	0.36
Diabetes (PC5)	0.001	0.47	0.53
Inflammation (PC3)	0.02	0.74	0.25
Inflammation (PC4)	0.01	0.72	0.27
Alzheimers (PC6)	0.007	0.88	0.11
Aging (PC2)	0.001	0.49	0.50
Aging (PC4)	0.006	0.66	0.33

Table S4 - Ranking measurement occasions by their importance (i.e. their relative magnitude) for PCs (with credible lifetime SES coefficients) including marital status as covariate.

Signatures	Ranking	Posterior Probability
Lupus (PC5)	SES in young adulthood > Parental SES and SES in mid-adulthood	0.87
CVD (PC1)	Parental SES > SES in young and mid-adulthood	0.69
Colorectal (PC3)	Parental SES > SES in young and mid-adulthood	0.73
RA (PC1)	Parental SES > SES in young and mid-adulthood	0.94
RA (PC4)	SES in young adulthood > Parental SES and SES in mid-adulthood	0.83
RA (PC5)	Parental SES and SES in young adulthood> SES in mid-adulthood	0.43
Asthma (PC2)	SES in young adulthood > Parental SES and SES in mid-adulthood	0.68
Asthma (PC3)	Parental SES > SES in young and mid-adulthood	0.76
Diabetes (PC3)	Parental SES > SES in young and mid-adulthood	0.83
Diabetes (PC5)	SES in young adulthood > Parental SES and SES in mid-adulthood	0.88

**Signatures**	**Ranking**	**Posterior Probability**
Inflammation (PC3)	SES in young adulthood > Parental SES and SES in mid-adulthood	0.68
Inflammation (PC4)	SES in young adulthood > Parental SES and SES in mid-adulthood	0.88
Alzheimers (PC6)	Parental SES and SES in young adulthood > SES in mid-adulthood	0.88
Aging (PC2)	Parental SES > SES in young and mid-adulthood	0.96
Aging (PC4)	SES in young adulthood > Parental SES and SES in mid-adulthood	0.91

Appendix 7

Table S5 – Probability of regions of practical equivalence (ROPEs) for three broad life-course models based on Chumbley et al. (2021) including marital status as covariate and updated imputation procedure.

Signatures	Accumulation	Sensitive	Critical
CVD (PC1)	0.009000	0.702500	0.288500
Lupus (PC5)	0.009625	0.667500	0.322875
Colorectal (PC3)	0.006375	0.773875	0.219750
RA (PC1)	0.001250	0.461125	0.537625
RA (PC4)	0.006500	0.609875	0.383625
RA (PC5)	0.026125	0.844125	0.129750
Asthma (PC2)	0.019250	0.735500	0.245250
Asthma (PC3)	0.007750	0.680625	0.311625
Diabetes (PC3)	0.004125	0.535000	0.460875
Diabetes (PC5)	0.002000	0.476375	0.521625
Inflammation (PC2)	0.001500	0.367875	0.630625
Inflammation (PC3)	0.018500	0.737375	0.244125
Inflammation (PC4)	0.011000	0.707875	0.281125
Alzheimers (PC6)	0.009000	0.901000	0.090000
Aging (PC2)	0.000500	0.393625	0.605875
Aging (PC4)	0.005375	0.665250	0.329375

Table S6 - Ranking measurement occasions by their importance (i.e. their relative magnitude) for PCs (with credible lifetime SES coefficients) including marital status as covariate and updated imputation procedure.

Signatures	Ranking	Posterior Probability
Lupus (PC5)	SES in young adulthood > Parental SES and SES in mid-adulthood	0.856250
RA (PC4)	SES in young adulthood > Parental SES and SES in mid-adulthood	0.804625
Asthma (PC2)	SES in young adulthood > Parental SES and SES in mid-adulthood	0.654875
Diabetes (PC5)	SES in young adulthood > Parental SES and SES in mid-adulthood	0.845750
Inflammation (PC3)	SES in young adulthood > Parental SES and SES in mid-adulthood	0.672875
Inflammation (PC4)	SES in young adulthood > Parental SES and SES in mid-adulthood	0.873750

Signatures	Ranking	Posterior Probability
Aging (PC4)	SES in young adulthood > Parental SES and SES in mid-adulthood	0.900250
CVD (PC1)	Parental SES > SES in young and mid-adulthood	0.809000
Colorectal (PC3)	Parental SES > SES in young and mid-adulthood	0.856000
RA (PC1)	Parental SES > SES in young and mid-adulthood	0.975125
Asthma (PC3)	Parental SES > SES in young and mid-adulthood	0.830250
Diabetes (PC3)	Parental SES > SES in young and mid-adulthood	0.894875
Inflammation (PC2)	Parental SES > SES in young and mid-adulthood	0.943625
Aging (PC2)	Parental SES > SES in young and mid-adulthood	0.985375
Alzheimers (PC6)	Parental SES and SES in young adulthood> SES in mid-adulthood	0.894250
RA (PC5)	Parental SES and SES in young adulthood> SES in mid-adulthood	0.468875

Table S7 – Decomposition of the weighted total effects in Average Direct Effect (ADE) and Average Causal Mediated Effects (ACME) with credible interval for each of the PCs including marital status as covariate and updated imputation procedure.

**Signatures**	**ADE**	**ACME**	**CrI Low**	**CrI High**	**Proportion Mediated**
CVD (PC1)	0.013	0.008	0.005	0.012	0.379
Lupus (PC5)	0.016	0.018	0.013	0.023	0.525
Colorectal (PC3)	0.026	0.008	0.005	0.012	0.244
RA (PC1)	0.019	0.008	0.005	0.012	0.302
RA (PC4)	0.023	0.006	0.003	0.009	0.190
RA (PC5)	0.021	0.007	0.003	0.011	0.239
Asthma (PC2)	0.029	0.007	0.004	0.011	0.191
Asthma (PC3)	0.024	0.002	-0.001	0.005	0.067
Diabetes (PC3)	0.009	0.005	0.003	0.008	0.355
Diabetes (PC5)	0.013	0.010	0.007	0.014	0.428
Inflammation (PC2)	-0.020	-0.005	-0.008	-0.002	0.188
Inflammation (PC3)	0.013	0.007	0.005	0.010	0.354
Inflammation (PC4)	0.026	0.015	0.011	0.020	0.367
Alzheimers (PC6)	0.019	0.010	0.007	0.014	0.340
Aging (PC2)	0.025	0.003	0.001	0.006	0.111
Aging (PC4)	0.034	0.013	0.009	0.018	0.274

Appendix 8

Table S8 – Probability of regions of practical equivalence (ROPEs) for three broad life-course models based on Chumbley et al. (2021) including different set of prior (Cauchy).

Signatures	Accumulation	Sensitive	Critical
CVD (PC1)	0.010375	0.713125	0.276500
Lupus (PC5)	0.008375	0.662250	0.329375
Colorectal (PC3)	0.008375	0.772375	0.219250
RA (PC1)	0.000875	0.461375	0.537750
RA (PC4)	0.007375	0.614750	0.377875
RA (PC5)	0.026625	0.832875	0.140500
Asthma (PC2)	0.017125	0.728250	0.254625
Asthma (PC3)	0.007750	0.686875	0.305375
Diabetes (PC3)	0.003375	0.549625	0.447000
Diabetes (PC5)	0.003000	0.475375	0.521625
Inflammation (PC2)	0.000875	0.373625	0.625500
Inflammation (PC3)	0.019000	0.739125	0.241875
Inflammation (PC4)	0.013875	0.699125	0.287000
Alzheimers (PC6)	0.008250	0.880375	0.111375
Aging (PC2)	0.000500	0.389125	0.610375
Aging (PC4)	0.006125	0.651750	0.342125

Table S9 - Ranking measurement occasions by their importance (i.e. their relative magnitude) for PCs (with credible lifetime SES coefficients)) including different prior (Cauchy).

Signatures	Ranking	Posterior Probability
Lupus (PC5)	SES in young adulthood > Parental SES and SES in mid-adulthood	0.853625
RA (PC4)	SES in young adulthood > Parental SES and SES in mid-adulthood	0.806375
Asthma (PC2)	SES in young adulthood > Parental SES and SES in mid-adulthood	0.645625
Diabetes (PC5)	SES in young adulthood > Parental SES and SES in mid-adulthood	0.848500
Inflammation (PC3)	SES in young adulthood > Parental SES and SES in mid-adulthood	0.663750
Inflammation (PC4)	SES in young adulthood > Parental SES and SES in mid-adulthood	0.866875

Signatures	Ranking	Posterior Probability
Aging (PC4)	SES in young adulthood > Parental SES and SES in mid-adulthood	0.894250
CVD (PC1)	Parental SES > SES in young and mid-adulthood	0.798125
Colorectal (PC3)	Parental SES > SES in young and mid-adulthood	0.856625
RA (PC1)	Parental SES > SES in young and mid-adulthood	0.974625
Asthma (PC3)	Parental SES > SES in young and mid-adulthood	0.826000
Diabetes (PC3)	Parental SES > SES in young and mid-adulthood	0.890750
Inflammation (PC2)	Parental SES > SES in young and mid-adulthood	0.950000
Aging (PC2)	Parental SES > SES in young and mid-adulthood	0.986125
RA (PC5)	Parental SES and SES in young adulthood> SES in mid-adulthood	0.465125
Alzheimers (PC6)	Parental SES and SES in young adulthood> SES in mid-adulthood	0.873250

References

Alrashoudi, Reem H., Isabel J. Crane, Heather M. Wilson, Monther Al-Alwan, and Nehad M. Alajez. 2018. "Gene Expression Data Analysis Identifies Multiple Deregulated Pathways in Patients with Asthma." *Bioscience Reports* 38 (6): 1–11. https://doi.org/10.1042/BSR20180548.

Baechler, Emily C., Franak M. Batliwalla, George Karypis, Patrick M. Gaffney, Ward A. Ortmann, Karl J. Espe, Katherine B. Shark, et al. 2003. "Interferon-Inducible Gene Expression Signature in Peripheral Blood Cells of Patients with Severe Lupus." *Proceedings of the National Academy of Sciences of the United States of America* 100 (5): 2610–15. https://doi.org/10.1073/pnas.0337679100.

Bahr, Timothy M., Grant J. Hughes, Michael Armstrong, Rick Reisdorph, Christopher D. Coldren, Michael G. Edwards, Christina Schnell, et al. 2013. "Peripheral Blood Mononuclear Cell Gene Expression in Chronic Obstructive Pulmonary Disease." *American Journal of Respiratory Cell and Molecular Biology* 49 (2): 316–23. https://doi.org/10.1165/rcmb.2012-0230OC.

Bull, Todd M., Christopher D. Coldren, Mark Moore, Sylk M. Sotto-Santiago, David V. Pham, S. Patrick Nana-Sinkam, Norbert F. Voelkel, and Mark W. Geraci. 2004. "Gene Microarray Analysis of Peripheral Blood Cells in Pulmonary Arterial Hypertension." *American Journal of Respiratory and Critical Care Medicine* 170 (8): 911–19. https://doi.org/10.1164/rccm.200312-1686OC.

Bürkner, Paul-Christian. 2017. "Brms : An R Package for Bayesian Multilevel Models Using Stan." *Journal of Statistical Software* 80 (August). https://doi.org/10.18637/jss.v080.i01.

Guinney, Justin, Rodrigo Dienstmann, Xin Wang, Aurélien De Reyniès, Andreas Schlicker, Charlotte Soneson, Laetitia Marisa, et al. 2015. "The Consensus Molecular Subtypes of Colorectal Cancer." *Nature Medicine* 21 (11): 1350–56. https://doi.org/10.1038/nm.3967.

Hauser, Robert M., and John Robert Warren. 1997. "Socioeconomic Indexes for Occupations: A Review, Update, and Critique." *Sociological Methodology* 27 (1): 177–298. https://doi.org/10.1111/1467-9531.271028.

Hout, Michael, Tom W Smith, and Peter V Marsden. 2014. "Prestige and Socioeconomic Scores for the 2010 Census Codes." *GSS Methodological Report No. 124*, no. 124, 1–18.

Leek, Jeffrey T., W. Evan Johnson, Hilary S. Parker, Andrew E. Jaffe, and John D. Storey. 2012. "The Sva Package for Removing Batch Effects and Other Unwanted Variation in High-Throughput Experiments." *Bioinformatics* 28 (6): 882–83. https://doi.org/10.1093/bioinformatics/bts034.

Loza, Matthew J., Charles E. McCall, Liwu Li, William B. Isaacs, Jianfeng Xu, and Bao Li Chang. 2007. "Assembly of Inflammation-Related Genes for Pathway-Focused Genetic Analysis." *PLoS ONE* 2 (10). https://doi.org/10.1371/journal.pone.0001035.

Nikpay, Majid, Anuj Goel, Hong Hee Won, Leanne M. Hall, Christina Willenborg, Stavroula Kanoni, Danish Saleheen, et al. 2015. *Nature Genetics* 47 (10): 1121–30. https://doi.org/10.1038/ng.3396.

Olsen, N. J., T. Sokka, C. L. Seehorn, B. Kraft, K. Maas, J. Moore, and T. M. Aune. 2004. "A Gene Expression Signature for Recent Onset Rheumatoid Arthritis in Peripheral Blood Mononuclear Cells." *Annals of the Rheumatic Diseases* 63 (11): 1387–92. https://doi.org/10.1136/ard.2003.017194.

Peters, Marjolein J., Roby Joehanes, Luke C. Pilling, Claudia Schurmann, Karen N. Conneely, Joseph Powell, Eva Reinmaa, et al. 2015. "The Transcriptional Landscape of Age in Human Peripheral Blood." *Nature Communications* 6. https://doi.org/10.1038/ncomms9570.

Robinson, Mark D., Davis J. McCarthy, and Gordon K. Smyth. 2009. "edgeR: A Bioconductor Package for Differential Expression Analysis of Digital Gene Expression Data." *Bioinformatics* 26 (1): 139–40. https://doi.org/10.1093/bioinformatics/btp616.

Sood, Sanjana, Iain J. Gallagher, Katie Lunnon, Eric Rullman, Aoife Keohane, Hannah Crossland, Bethan E. Phillips, et al. 2015. "A Novel Multi-Tissue RNA Diagnostic of Healthy Ageing Relates to Cognitive Health Status." *Genome Biology* 16 (1): 1–17. https://doi.org/10.1186/s13059-015-0750-x.

Tibshirani, Daniela Witten and Rob. 2020. "PMA: Penalized Multivariate Analysis." https://cran.r-project.org/web/packages/PMA/index.html.

Wang, Yulei, Catalin C. Barbacioru, Dov Shiffman, Sriram Balasubramanian, Olga Iakoubova, Maryann Tranquilli, Gonzalo Albornoz, et al. 2007. "Gene Expression Signature in Peripheral Blood Detects Thoracic Aortic Aneurysm." *PLoS ONE* 2 (10). https://doi.org/10.1371/journal.pone.0001050.

Xue, Angli, Yang Wu, Zhihong Zhu, Futao Zhang, Kathryn E. Kemper, Zhili Zheng, Loic Yengo, et al. 2018. "Genome-Wide Association Analyses Identify 143 Risk Variants and Putative Regulatory Mechanisms for Type 2 Diabetes." *Nature Communications* 9 (1): 2941. https://doi.org/10.1038/s41467-018-04951-w.

Zhuang, Haotian, Huimin Wang, and Zhicheng Ji. 2022. "findPC: An R Package to Automatically Select the Number of Principal Components in Single-Cell Analysis." *Bioinformatics* 38 (10): 2949–51. https://doi.org/10.1093/bioinformatics/btac235.
